# Critical Factors in Human Antizymes that Determine the Differential Binding, Inhibition, and Degradation of Human Ornithine Decarboxylase

**DOI:** 10.3390/biom9120864

**Published:** 2019-12-12

**Authors:** Ju-Yi Hsieh, Yen-Chin Liu, I-Ting Cheng, Chu-Ju Lee, Yu-Hsuan Wang, Yi-Shiuan Fang, Yi-Liang Liu, Guang-Yaw Liu, Hui-Chih Hung

**Affiliations:** 1 Department of Life Sciences and Institute of Genomics & Bioinformatics, National Chung Hsing University, Taichung 40227, Taiwan; crab882000@gmail.com (J.-Y.H.); poooorme@gmail.com (Y.-C.L.); etta0624@gmail.com (I.-T.C.); shirly30020@yahoo.com.tw (C.-J.L.); wmaluco0527@gmail.com (Y.-H.W.); tw7629@gmail.com (Y.-S.F.); sgl@dragon.nchu.edu.tw (Y.-L.L.); 2 Institute of Biochemistry, Microbiology & Immunology, Chung Shan Medical University, Taichung 40201, Taiwan; 3 Division of Allergy, Immunology, and Rheumatology, Chung Shan Medical University Hospital, Taichung 40201, Taiwan; 4 iEGG & Animal Biotechnology Center, National Chung Hsing University, Taichung 40227, Taiwan

**Keywords:** protein–protein interaction, ubiquitin-independent degradation, binding affinity, AZ isoform

## Abstract

Antizyme (AZ) is a protein that negatively regulates ornithine decarboxylase (ODC). AZ achieves this inhibition by binding to ODC to produce AZ-ODC heterodimers, abolishing enzyme activity and targeting ODC for degradation by the 26S proteasome. In this study, we focused on the biomolecular interactions between the C-terminal domain of AZ (AZ_95–228_) and ODC to identify the functional elements of AZ that are essential for binding, inhibiting and degrading ODC, and we also identified the crucial factors governing the differential binding and inhibition ability of AZ isoforms toward ODC. Based on the ODC inhibition and AZ-ODC binding studies, we demonstrated that amino acid residues reside within the α1 helix, β5 and β6 strands, and connecting loop between β6 and α2 (residues 142–178), which is the posterior part of AZ_95–228_, play crucial roles in ODC binding and inhibition. We also identified the essential elements determining the ODC-degradative activity of AZ; amino acid residues within the anterior part of AZ_95–228_ (residues 120–145) play crucial roles in AZ-mediated ODC degradation. Finally, we identified the crucial factors that govern the differential binding and inhibition of AZ isoforms toward ODC. Mutagenesis studies of AZ1 and AZ3 and their binding and inhibition revealed that the divergence of amino acid residues 124, 150, 166, 171, and 179 results in the differential abilities of AZ1 and AZ3 in the binding and inhibition of ODC.

## 1. Introduction

Antizyme (AZ) is a central player in the regulatory circuit that controls cellular levels of polyamines by regulating ornithine decarboxylase (ODC) degradation and polyamine uptake activity [1,2,3,4]. AZ was originally identified as a negative regulatory protein of ODC because it facilitates the degradation of ODC [1,2,3]. ODC is the first and rate-limiting enzyme in polyamine biosynthesis and a central regulator of cellular polyamine synthesis [3,4,5,6]. Because ODC and cellular polyamines have important roles in numerous biological functions, such as embryonic development; the cell cycle, proliferation, differentiation, and apoptosis, the activity and half-life of ODC are tightly controlled [7,8,9,10,11,12]. In fact, ODC undergoes a unique ubiquitin-independent and AZ-promotable protein degradation by the 26S proteasome via a direct interaction with AZ [13]. AZ binding promotes the dissociation of ODC homodimers to form AZ-ODC heterodimers, abolishing ODC enzyme activity and targeting ODC for degradation by the 26S proteasome [3,14,15,16,17,18,19,20,21]. Another regulatory protein, antizyme inhibitor (AZI), is homologous to the ODC enzyme; it shows stronger binding to AZ than ODC, thus releasing ODC from the ODC-AZ complex, restoring ODC activity via antagonizing the AZ function [17,18,19] and preventing the proteolytic degradation of ODC. In addition to interacting with ODC and AZI, AZ has been shown to bind to and facilitate the AZ-dependent degradation of cell cycle-regulating proteins, including cyclin D1, Aurora-A kinase and Smad1 [22,23,24,25,26]. AZ functions as a negative regulator of cell growth and as a tumor suppressor. This protein represses cancer cell proliferation and the progression of neoplastic diseases through inhibiting ODC activity and polyamine transport and facilitating protein degradation of growth regulatory molecules, such as ODC, cyclin D1, and Aurora-A kinase [3,4,7,22,23,24,25,26,27,28,29,30,31].

AZ was the first protein shown to utilize translational frame shifting to regulate mammalian mRNA [32,33]. Polyamines stimulate a ribosomal translational frameshifting of AZ mRNA, causing the ribosome to bypass the first open reading frame (ORF) of AZ and allowing a fully functional 22-kDa AZ protein to be synthesized from the second ORF (+1 frameshift), which ultimately produces functional AZ proteins [34,35,36,37,38]. To date, at least three AZ isoforms with different binding affinities for ODC have been identified. AZ1 is present in all tissues and is the major isoform that participates in ODC degradation. The C-terminal region of AZ1 interacts with ODC to inhibit enzyme activity, and the middle region near the N-terminus of AZ1 controls the degradation of ODC [14,39,40]. We previously determined that the C-terminal region of AZ1 is essential and fully functional for the binding, inhibition, and degradation of ODC [40,41] and that the N-terminal region of AZ1 is the putative binding site of cyclin D1 [23]. Therefore, ODC and cyclin D1 bind to different regions of AZ1 [23]. AZ2 is also distributed in all tissues and cells, but its expression level is much lower than that of AZ1 [42]. Although AZ2 can inhibit ODC enzyme activity, it does not promote ODC degradation, suggesting that it acts as a reversible storage compartment to stabilize the ODC monomer by forming a heterodimer [43,44]. AZ3 is specific to the germ cells of testes, where expression is restricted to the post-meiotic stage of spermatogenesis during the differentiation of male germ cells to mature sperm [45]. AZ3 inhibition of ODC activity is weaker than that of AZ1; this protein fails to stimulate ODC degradation but is similar to AZ1 and AZ2 in polyamine transportation [46].

**Figure 1:**
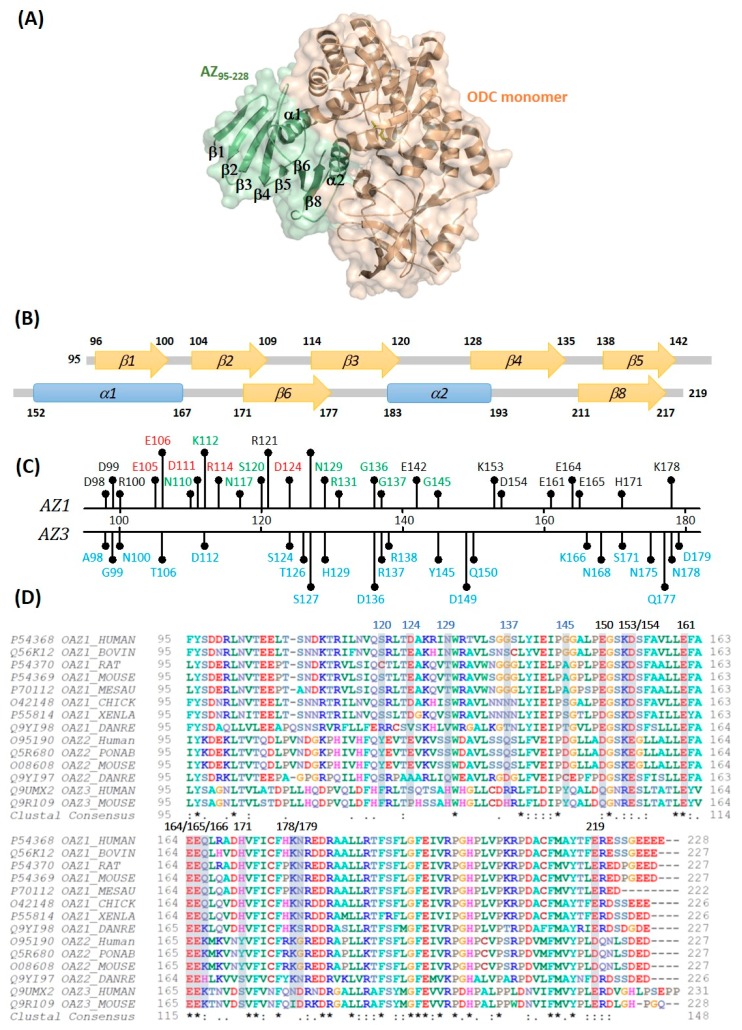
Crystal structure of the human AZ_95–228_-ODC heterodimer. (**A**) Complex structure of the AZ_95–228_-ODC heterodimer (PDB: 4ZGY); this figure was generated using PyMOL [47]. (**B**) Secondary structure of the human AZ_95–228_ peptide consists of two α-helices and eight β-strands and their connecting loops. (**C**) Amino acid residues for the mutagenesis study. Amino acid residues colored in black were AZ1 study for ODC binding and inhibition, colored in green were AZ1 study for ODC degradation, and colored in red were AZ1 study for ODC inhibition and degradation; while amino acid residues colored in blue were AZ3 study for ODC binding and inhibition. (**D**) Multiple sequence alignments of AZ isoforms. The multiple sequence alignment was generated by ClustalW2 [48].

Our previous studies have shown that the N-terminus of AZ is not essential for the binding and inhibition of ODC, and the C-terminal AZ peptide from residues 95 to 228 (AZ_95–228_) was sufficient for binding and inhibiting ODC [40]. We also resolved the crystal structure of the human AZ_95–228_-ODC complex (Figure 1A) and found that AZ_95–228_ displays eight β-strands and two α-helices (Figure 1B). Because AZ_95–228_ was also sufficient for ODC degradation [41], the AZ_95–228_-ODC complex may be the core structure for 26S proteasomal recognition. In this study, we focused on the biomolecular interactions between the C-terminal domain of AZ1 (AZ_95–228_) and ODC to identify the functional elements of AZ that are essential for binding, inhibiting and degrading ODC. Furthermore, we identified the crucial factors that govern the differential binding and inhibition of AZ1 and AZ3 toward ODC.

## 2. Materials and Methods

### 2.1. Expression and Purification of Recombinant ODC and AZ

Human ODC, AZ, and a series of AZ mutant proteins were subcloned into an N-terminal His6-tagged pQE30 vector (Qiagen, Hilden, Germany). The expression vector with ODC or AZ gene was introduced to JM109 *Escherichia coli* (Agilent, Palo Alto, CA, USA) to express the target proteins. ODC or AZ proteins were overexpressed with 1 mM isopropyl-1-thio-β-D-galactoside (IPTG) induction in JM109 cells for 20 h at 25 °C. After harvesting the cells, the total cell extract was applied to a His-Select™ nickel affinity column (Sigma, St. Louis, MO, USA) for further purification. The protocol for the protein purification of ODC or AZ was followed as described in Hsieh et al. [40]. First, discarded proteins in the lysate-Ni-NTA mixture were washed out using a buffer containing 10 mM imidazole, 500 mM NaCl, 2 mM β-mercaptoethanol and 30 mM Tris-HCl at pH 7.6. Subsequently, the target protein was eluted out with an elution buffer containing 250 mM imidazole, 500 mM NaCl, 30 mM Tris-HCl, and 2 mM β-mercaptoethanol (pH 7.6). Finally, the protein purity was examined by 10% sodium dodecyl sulfate polyacrylamide gel electrophoresis (SDS-PAGE).

### 2.2. Site-Directed Mutagenesis

Site-directed mutagenesis on AZ1 or AZ3 was carried out with a QuikChange™ kit to generate the AZ1 and AZ3 mutants (Agilent, Palo Alto, CA, USA). The mutagenic primers with the desired mutations were approximately 35–45 bases. The mutations in this study are shown in Table S2. A polymerase chain reaction (PCR) using Pfu DNA polymerase was performed a total of 18–20 cycles to amplify the mutagenic DNA. The PCR product was treated with DpnI to digest the unwanted wild-type DNA, then the DNA with the specific mutation was transformed into the XL 10-Gold (Agilent, Palo Alto, CA, USA) *E. coli* strain. Finally, the DNA sequence with the desired mutation was confirmed by auto sequencing. 

### 2.3. Assay of ODC Activity in the Presence of AZ

The continuous ODC enzyme activity was measured by the reactions that were coupled with the phosphoenolpyruvate carboxylase and malate dehydrogenase, and the ODC enzyme (0.38 μM) was inhibited with various quantities of AZ proteins [17]. The assay mixture in a final volume of 0.5 mL contained 30 mM Tris-HCl at pH 7.4, 10 mM ornithine, 0.02 mM pyridoxal 5′-pyrophosphate, and 0.4 mL of the CO2-L3K assay kit solution (DCL, Charlottetown, Canada), which had 12.5 mM PEP, >0.4 U/mL phosphoenolpyruvate carboxylase (microbial), >4.1 U/mL malate dehydrogenase (mammalian), and 0.6 mM NADH analog. 

The reaction was traced at the absorbance decrease at 405 nm using a Perkin–Elmer Lamba-25 spectrophotometer, and the production of 1 mmol of CO_2_ was accompanied by the oxidation of 1 mmol of NADH analog in this coupled reaction. For the NADH analog, an extinction coefficient of 2410 cm^−1^ mM^−1^ was used in the calculations. The IC_50_ value of each inhibition plot was calculated with the following equation:

ODC enzyme activity = A + (B − A)/[1 + ([AZ]/IC_50_) ^Hill slope^) (1)

where A and B are the minimum and maximum ODC enzyme activity, respectively, and the Hill slope provides the largest slope of the curve. The IC_50_ value denotes the AZ concentration that is required for inhibiting 50% of the ODC enzyme activity. All calculations were carried out with the SigmaPlot 10.0 software program (Jandel, San Rafael, CA, USA).

### 2.4. Analysis of Size Distributions of the AZ-ODC Heterodimer by Analytical Ultracentrifugation

Sedimentation velocity experiments were carried out at 20 °C with a Beckman Optima XL-A analytical ultracentrifuge. In a buffer of 30 mM Tris-HCl (pH 7.4) and 25 mM NaCl, the concentration of ODC was fixed at 0.3 mg/mL with AZ concentrations AZ ranging from 0.02 to 0.19 mg/mL (the molar ratio of AZ/ODC ranged from 0.25 to 3). In the centerpiece, the reference and sample sectors were filled with buffer (400 μL) and sample protein (380 μL), respectively, and the whole module was then set up in an An-50 Ti rotor. Sedimentation velocity experiments were implemented at 20 °C with a rotor speed of 42,000 rpm using the absorbance at 280 nm to measure every 420 s with a step size of 0.002 cm. At different time points, a total of 20–30 scans were collected and fitted to a continuous size distribution model with the program SEDFIT [49,50]. All size distributions were calculated from 0.1 to 20 s with a confidence level of *p* = 0.95 and a resolution N of 200.

To determine the dissociation constant (*K_d_*) of the AZ-ODC complexes, sedimentation velocity experiments were carried out with different concentrations of AZ proteins at a constant concentration of ODC protein. The *K_d_* values of the AZ-ODC complexes were calculated by totally fitting all data sets with the AB hetero-association model in the SEDPHAT program [51,52].

### 2.5. In Vitro Degradation Assay

AZ-ODC proteins at a fixed molar ratio (1:1) were incubated with a rabbit reticulocyte lysate (Promega, Madison, WI, USA) mixture including 40 mM Tris-HCl (pH 7.4), 5 mM MgCl_2_, 2 mM dithiothreitol (DTT), 1.5 mM ATP, 10 mM creatine phosphate (Sigma, St. Louis, MO, USA), and 1.6 mg/mL creatine phosphokinase (Sigma, St Louis, MO, USA) for 2 h at 37 °C. The degradation reaction was stopped after adding the 2× protein sample dye. After the proteins were separated by 13.5% SDS-PAGE, they were transferred to a polyvinylidene difluoride (PVDF) membrane for immunoblotting using anti-ODC (CUSABIO, Houston, TX, USA), anti-AZ (MDBio, Taiwan), and anti-GAPDH (GeneTex, Irvine, CA, USA) antibodies as probes, and the protein bands of ODC, AZ, and GAPDH (internal control) were monitored by ImageQuant™ LAS 4000 (GE Healthcare, Boston, MA, USA). The degradation ratio of ODC at 2 h was quantitated by the program ImageJ when the amount of ODC at 0 h was defined as 1 [53].

## 3. Results and Discussion

### 3.1. Inhibitory Effect of the AZ_95–228_ Mutants on ODC

Based on the crystal structure of the ODC-AZ_95–228_ heterodimer, we selected 18 positively or negatively charged amino acid residues in the C-terminal domain of AZ1 (AZ_95–228_) to bind and inhibit ODC (Table 1 and Table S1). The residues Asp98, Asp99, Arg100, Glu105, Glu106, Asp111, Arg114, Arg121, and Asp124 are located in the β1 *to* β3 strands and connecting loops between these β strands; Glu142 is located at the end of β5; Lys153, Asp154, Glu161, Glu164, and Glu165 are part of the α1 helix; His171 is located at the beginning of β6 and Lys178 is located on the loop between β6 and α2. Figure 1B indicates the location of each amino acid residue.

In the ODC inhibition studies, the AZ_95–228_ single mutants displayed differential inhibition of ODC (Table 1 and Table S1). The inhibitory effects of AZ_95–228__D98A, AZ_95–228__D99A, AZ_95–228__R100A, AZ_95–228__E105A, AZ_95–228__E106A, AZ_95–228__D111A, AZ_95–228__R114A, AZ_95–228__R121A, and AZ_95–228__D124A were similar to that of AZ_95–228_ (Figure S1). The IC_50_ values of these mutants were approximately 0.16~0.20 μM, and the IC_50_ value of AZ_95–228__WT was 0.16 μM (Table S1); the IC_50_-fold (IC_50,mutant_/IC_50,WT_) values of these mutants were approximately 1~1.3-fold. In contrast, the IC_50_ values of AZ_95–228__E142A, AZ_95–228__K153A, AZ_95–228__D154A, AZ_95–228__E161A, AZ_95–228__E164A, AZ_95–228__D165A, AZ_95–228__H171A, and AZ_95–228__K178A were higher than those of AZ_95–228__WT (Table 1), and the inhibitory effects of these mutants were weaker than that of AZ_95–228_ (Figure S2). The IC_50_ values of these mutants were approximately 0.25~0.4 μM (Table 1), and the IC_50_-fold values (IC_50,mutant_/IC_50,WT_) were approximately 1.6~2.5-fold.

**Figure 2:**
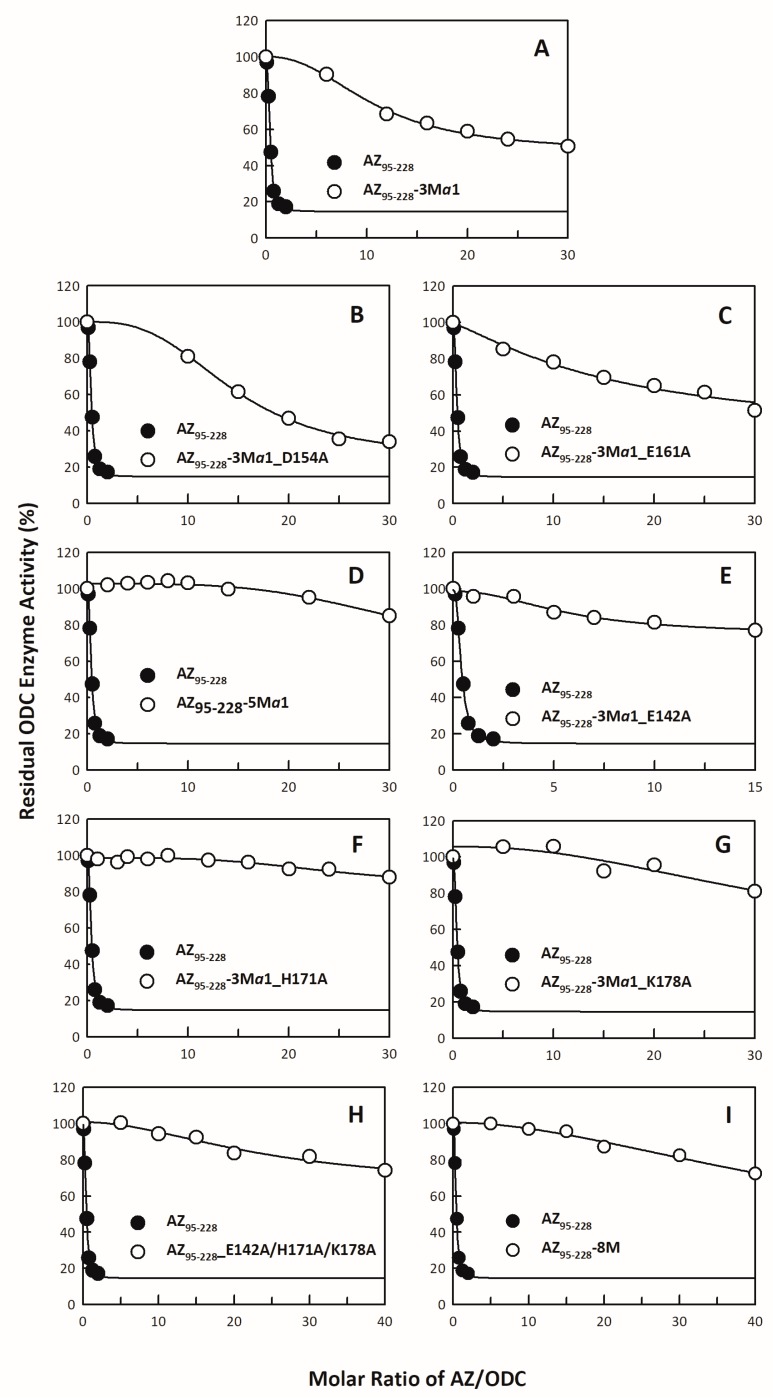
Inhibition plots of the ODC enzyme with multiple mutants of AZ_95–228_. The enzyme activity of ODC was inhibited by various multiple mutants of AZ_95–228_. The IC_50_ values of multiple mutants of AZ_95–228_ presented in Table 1 were derived by curve-fitting the inhibition plots. The molar ratio refers to AZ versus the ODC monomer. (**A**) AZ_95–228__K153A/E164A/E165A (AZ_95–228_-3Mα1), (**B**) AZ_95–228_-3Mα1_D154A, (**C**) AZ_95–228_-3Mα1_E161A, (**D**) AZ_95–228__K153A/D154A/E161A/E164A/E165A (AZ_95–228_-5Mα1), (**E**) AZ_95–228_-3Mα1_E142A, (**F**) AZ_95–228_-3Mα1_H171A, (**G**) AZ_95–228_-3Mα1_K178A, (**H**) AZ_95–228__E142A/H171A/K178A, and (**I**) AZ_95–228__E142A/K153A/D154A/E161A/E164A/E165A/H171A/K178A (AZ_95–228_-8M).

Among these single mutants of the α1 helix, AZ_95–228__K153A, AZ_95–228__E164A, and AZ_95–228__E165A, which had mutations, had IC_50_ values greater than those of the other mutants (Table 1); therefore, a triple mutant, AZ_95–228__K153A/E164A/E165A (AZ_95–228_-3Mα1), was created to examine its ability to inhibit ODC (Figure 2A). The IC_50_ value of AZ_95–228_-3 Mα1 was 4.13 μM, 26-fold greater than that of AZ_95–228_ (Table 1). Two quadruple mutants, AZ_95–228_-3Mα1_D154A and AZ_95–228_-3Mα1_E161A, had weaker inhibitory effects than AZ_95–228_-3Mα1 (Figure 2B,C, respectively); therefore, a quintuple mutant of the α1 helix, AZ_95–228__K153A/D154A/E161A/E164A/E165A (AZ_95–228_-5Mα1), was generated, and this AZ_95–228_-5Mα1 mutant completely lost its inhibition of ODC enzyme activity (Figure 2D), clearly indicating the significance of the α1 helix for ODC inhibition.

In addition to the amino acid residues in the α1 region, residues on β5 (His142), β6 (His171), and connecting loop between β6 and α2 (Lys178) also play a role in ODC inhibition (Table 1). Adding E142A, H171A, or K178A to AZ_95–228_-3Mα1 strongly influenced AZ-induced ODC inhibition. The ability of AZ_95–228_-3Mα1_E142A, AZ_95–228_-3Mα1_H171A, and AZ_95–228_-3Mα1_K178A to inhibit ODC enzyme activity was completely lost (Figure 2E–G, respectively; Table 1). 

An α1 and non-α1 mutant, AZ_95–228_-5Mα1 and AZ_95–228__E142A/H171A/K178, respectively, and the hybrid octuple mutant AZ_95–228__E142A/K153A/D154A/E161A/E164A/E165A/H171A/K178A (AZ_95–228_-8M) had no inhibitory effects on ODC (Figure 2D,H,I, respectively; Table 1). These results demonstrated that in the AZ structure, the α1 helix was the key region responsible for inhibition, and the β5 and β6 strands and connecting loop between β6 and α2 also play crucial roles in ODC inhibition, while the β1 *to* β3 strands and their connecting loops were not important for AZ-induced ODC inhibition.

### 3.2. Binding Affinity of the AZ_95–228_ Mutants toward ODC

**Figure 3:**
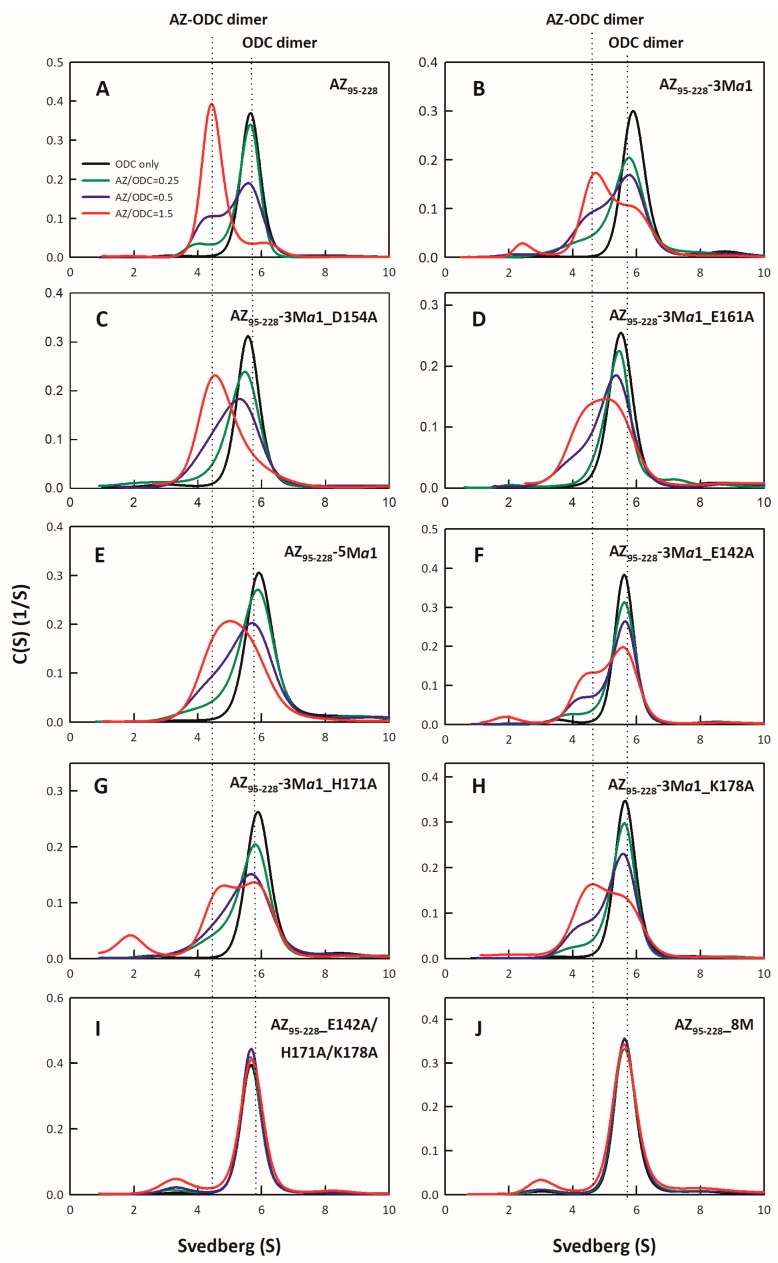
Size distribution plots of multiple mutants of AZ_95–228_-ODC heterodimers. (**A**) AZ_95–228_-ODC, (**B**) AZ_95–228_-3Mα1-ODC, (**C**) AZ_95–228_-3Mα1_D154A-ODC, (**D**) AZ_95–228_-3Mα1_E161A-ODC, (**E**) AZ_95–228_-5Mα1-ODC, (**F**) AZ_95–228_-3Mα1_E142A-ODC, (**G**) AZ_95–228_-3Mα1_H171A-ODC, (**H**) AZ_95–228_-3Mα1_K178A-ODC, (**I**) AZ_95–228__E142A/H171A/K178A-ODC, and (**J**) AZ_95–228_-8M-ODC. The sedimentation velocity data in each figure were globally fitted with the SEDPHAT program to acquire *K_d_* values for the AZ_95–228_-ODC heterodimers shown in Table 2.

AZ binds to ODC and dissociates the ODC dimer to form AZ-ODC heterodimers [17,18,19]. Here, we examined the binding affinities of the AZ_95–228_ mutants whose IC_50_-fold values (IC_50,mutant_/IC_50,WT_) exceeded 1.5 (Table 1). To determine the binding affinities of the AZ_95–228_ mutants toward ODC, we performed multiple size distribution analyses of AZ_95–228_-ODC heterodimers (Figure 3 and Figure S3), and all sedimentation data for each set were globally fitted to the AB hetero-association model in the SEDPHAT program to acquire the dissociation constant (*K_d_*) between AZ_95–228_ and ODC (Table 2). AZ_95–228_ dissociated the dimeric ODC to form AZ_95–228_-ODC heterodimers with a *K_d_*_,AZ-ODC_ value of 1.15 μM (Table 2). The single mutants that had a high IC_50_ value also had a higher *K_d_*_,AZ-ODC_ value than AZ_95–228_ (Figure S3; Table 2), indicating that the lower the ODC-binding affinity of AZ, the lower the ODC inhibition by AZ was.

The ODC binding affinities of the AZ_95–228_ α1 and non-α1 multiple mutants, as well as the hybrid octuple mutant, were determined (Figure 3). The *K_d_*_,AZ-ODC_ value of the AZ_95–228_-3Mα1-ODC heterodimers was 6.58 μM, 5.7-fold greater than that of AZ_95–228_-ODC (Figure 3B; Table 2). However, the ODC binding affinity of the quadruple or quintuple mutants, AZ_95–228_-3Mα1_D154A, AZ_95–228_-3Mα1_E161A, and AZ_95–228_-5Mα1, was not further significantly decreased (Figure 3C–E, respectively; Table 2); the *K_d_*_,AZ-ODC_ value of AZ_95–228_-5Mα1-ODC was 6.81 μM, 5.9-fold greater than that of AZ_95–228_, which was similar to that of AZ_95–228_-3Mα1-ODC (Table 2).

The ODC binding affinities of the following quadruple mutants, AZ_95–228_-3Mα1_E142A, AZ_95–228_-3Mα1_H171A, and AZ_95–228_-3Mα1_K178A, were further examined (Figure 3F–H, respectively). The *K_d_*_,AZ-ODC_ values of these mutants were greater than those of AZ_95–228_-3Mα1-ODC (Table 2). Furthermore, the *K_d_*_,AZ-ODC_ value of the non-α1 mutant AZ_95–228__E142A/H171A/K178A was 11.49 μM, 10-fold greater than that of AZ_95–228_ (Figure 3I; Table 2). The *K_d_*_,AZ-ODC_ of the hybrid octuple mutant (AZ_95–228_-8M) was 12.44 μM, higher than that of all of the mutants of AZ_95–228_-ODC (Figure 3J; Table 2), which indicated that these amino acid residues cooperate to allow AZ to bind and inhibit ODC. Briefly, the AZ-ODC binding affinity data were consistent with the inhibition data, and in the AZ structure, the posterior part of AZ_95–228_, which comprises the α1 helix, β5 and β6 strands, and connecting loop between β6 and α2 (residues 142–178) were shown to play crucial roles in ODC binding and inhibition.

### 3.3. Identification the Essential Elements Determining the ODC-Degradative Activity of AZ

Since we found that the critical ODC-binding domain of AZ resides within the posterior part of AZ_95–228_, the determinants for ODC degradation induced by AZ may reside within the anterior part of AZ_95–228_.

**Figure 4:**
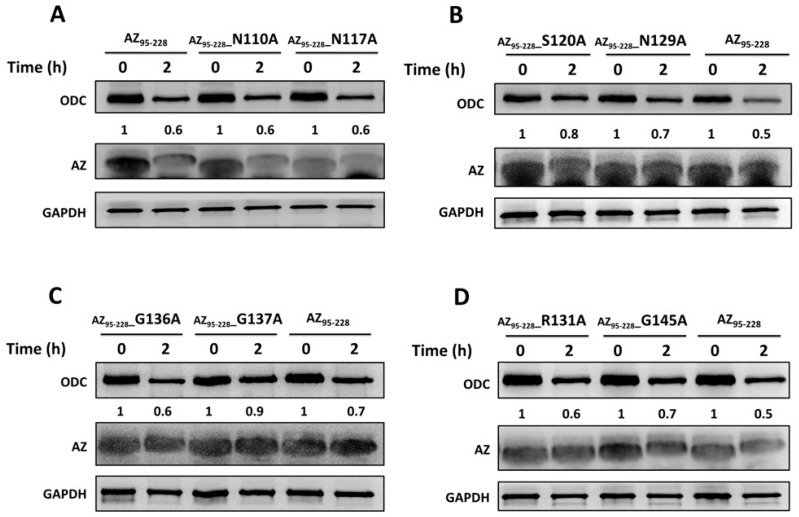
AZ-mediated ODC in vitro degradation with AZ mutant peptides in rabbit reticulocyte lysates. ODC can be effectively degraded by AZ binding, and protein degradation was detected by anti-ODC antibody (*n* = 3). (**A**) ODC degradation with AZ_95–228_, AZ_95–228__N110A, and AZ_95–228__N117A, (**B**) ODC degradation with AZ_95–228_, AZ_95–228__S120A and AZ_95–228__N129A, (**C**) ODC degradation with AZ_95–228_, AZ_95–228__G136A, and AZ_95–228__G137A, (**D**) ODC degradation with AZ_95–228_, AZ_95–228__R131A, and AZ_95–228__G145A. A residual amount of ODC protein at a different time was indicated under the ODC blotting gel in each figure.

An earlier study showed that the region containing residues 130–145 of AZ might be necessary for ODC degradation [44]. We identified some amino acid residues that are situated at residues 95–145 (Figure 1B) to examine their effect on ODC degradation. An AZ-mediated ODC degradation experiment with a series of AZ_34–228_ and AZ_95–228_ mutants were performed under a reticulocyte lysate-based system. Basically, AZ_34–228_ and AZ_95–228_ peptides had similar degradative activity toward ODC (Figure 4 and Figure S4). Mutations at charged amino acid residues, such as Glu105 and Glu106 in β2, Asn110, Asp111, and Lys112 in the loop between β2 and β3, Arg114 and Asn117 on β3, and Asp124 in the loop between β3 and β4, did not decrease the ODC-degradative activity; these AZ single mutants did not show retardation of ODC degradation (Figure 4A and Figure S4A–D). Nevertheless, mutation of these residues of AZ_95–228_, such as Ser120, Asn129, Gly137, and Gly145, seemed to cause the retardation of ODC degradation compared with that of AZ_95–228_, indicating that the ODC-degradative activity of the AZ_95–228__S120A, AZ_95–228__N129A, AZ_95–228__G137A, and AZ_95–228__G145A peptides was weaker than that of the AZ_95–228_ peptide (Figure 4B–D). The ODC-degradative activity of AZ_95–228__ R131A was not as evident as that of the AZ_95–228__G145A peptide (Figure 4D). Overall, in the AZ structure, Ser120 at the end of β3, Asn129 in β4, and Gly137 and Gly145 in the loops between β4 and β5 and between β5 and α1, respectively (Figure 1B), may govern the ODC-degradative activity of AZ; amino acid residues within the β1–β3 region and their connecting loops did not play a role in AZ-mediated ODC degradation. The anterior parts of AZ_95–228_ (residues 120–145) play crucial roles in AZ-mediated ODC degradation.

### 3.4. Identification of the Crucial Factors that Govern the Differential Binding and Inhibition of AZ Isoforms toward ODC

We have identified the amino acid residues in AZ, such as Glu142, Lys153, Asp154, Glu161, Glu164 and Glu165, His171 and Lys178, that function in the binding and inhibition of ODC (Table 1 and Table 2). Among these residues, only residues 171 and 178 are not conserved in AZ isoforms (Figure 1D). Analyses of AZ isoforms in the inhibition and binding studies showed that AZ1 and AZ2 had similar inhibition and binding of ODC, but AZ3 showed decreased inhibition and binding of ODC compared to AZ1 and AZ2 (Figure S5). Therefore, we further identified the determinants within the C-terminus of AZ1 and AZ3 that govern the differential binding and inhibitory effects toward ODC.

**Figure 5:**
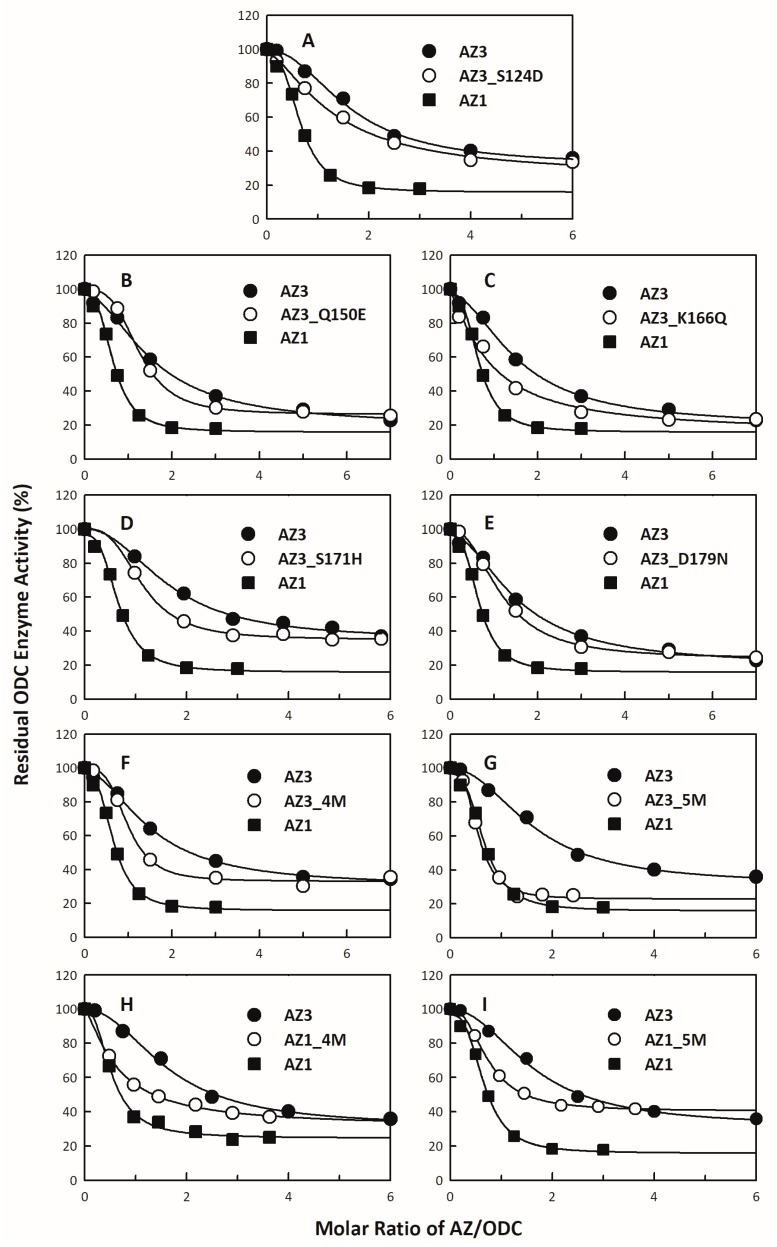
Inhibition plots of the ODC enzyme with single or multiple mutants of AZ1 and AZ3. The enzyme activity of ODC was inhibited by various single or multiple mutants of AZ1 or AZ3. The IC_50_ values of AZ1 or AZ3 mutants presented in Table 3 and Table 4 were derived by curve-fitting the inhibition plots. The molar ratio refers to AZ1 or AZ3 versus the ODC monomer. (**A**) AZ3_S124D, (**B**) AZ3_Q150E, (**C**) AZ3_K166Q, (**D**) AZ3_S171H, (**E**) AZ3_D179N, (**F**) AZ3_S124D/Q150E/K166Q/D179N (AZ3_4M), (**G**) AZ3_S124D/Q150E/K166Q/S171H/D179N (AZ3_5M), (**H**) AZ1_D124S/E150Q/Q166K/N179D (AZ1_4M), and (**I**) AZ1_D124S/E150Q/Q166K/H171S/N179D (AZ1_5M).

**Figure 6:**
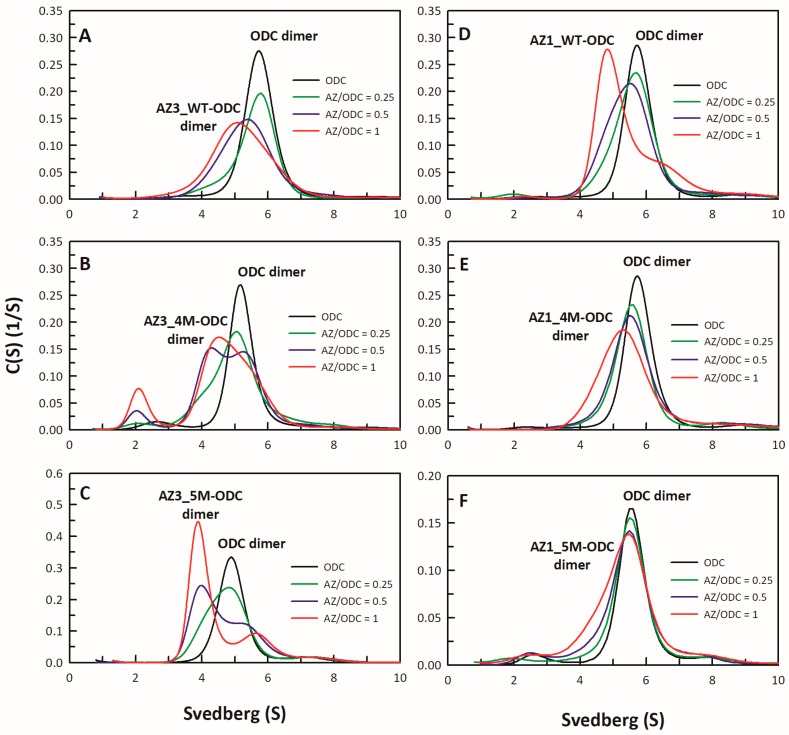
Size distribution plots of multiple mutants of AZ1-ODC or AZ3-ODC heterodimers. (**A**) AZ3_WT-ODC, (**B**) AZ3_4M-ODC, (**C**) AZ3_5M-ODC, (**D**) AZ1_WT-ODC, (**E**) AZ1_4M-ODC, and (**F**) AZ1_5M-ODC. The sedimentation velocity data in each figure were globally fitted with the SEDPHAT program to acquire *K_d_* values for the AZ-ODC heterodimers shown in Table 4.

To probe these differences, we changed the amino acid residues of AZ3 that are different from those of AZ1 to the amino acid residues found in AZ1. We examined the inhibitory effect of these single AZ3 mutants on ODC activities (Figure 5 and Figure S6; Table 3) and found that AZ3_S124D, AZ3_Q150E, AZ3_K166Q, AZ3_S171H, and AZ3_D179N had lower IC_50_ values than AZ3-WT (Figure 5A–E; Table 3). We further constructed quadruple and quintuple mutants of AZ3 to determine their abilities to inhibit and bind ODC. The IC_50_ values of AZ3_S124D/Q150E/K166Q/D179N (AZ3_4M) and AZ3_S124D/Q150E/K166Q/S171H/D179N (AZ3_5M) were 0.25 μM and 0.29 μM, respectively (Figure 5F,G, respectively; Table 4), and the *K_d_*_,AZ-ODC_ values of these two AZ3 mutants were 0.5 μM and 0.14 μM, respectively, notably smaller than those of AZ3_WT (Table 4). Both the IC_50_ and *K_d_*_,AZ-ODC_ values of AZ3_5M (0.29 μM and 0.14 μM, respectively) were reduced to levels similar to those of AZ1_WT (0.23 μM and 0.22 μM, respectively), indicating that AZ3_5M is similar to AZ1_WT in ODC binding and inhibition (Table 4). We also constructed AZ1 quadruple and quintuple mutants to determine their abilities to inhibit and bind ODC. The IC_50_ values of AZ1_D124S/E150Q/Q166K/N179D (AZ1_4M) and AZ1_D124S/E150Q/Q166K/N179D/H171S (AZ1_5M) were 0.55 μM and 0.49 μM, respectively (Figure 5H,I, respectively; Table 4), and the *K_d_*_,AZ-ODC_ values of these AZ1 mutants were 0.95 μM and 1.78 μM, respectively (Figure 6D,E, respectively; Table 4). Both the IC_50_ and *K_d_*_,AZ-ODC_ values of AZ1_5M (0.49 μM and 1.78 μM, respectively) were elevated to levels similar to those of AZ3_WT (0.61 μM and 1.52 μM, respectively), indicating that AZ1_5M is similar to AZ3_WT in ODC binding and inhibition (Table 4). These data indicated that the divergence of these amino acid residues governs the differential abilities of AZ1 and AZ3 to bind and inhibit ODC.

### 3.5. Structural Elements of AZ Responsible for Binding, Inhibition, and Degradation of ODC

We have shown that the C-terminal region of AZ from residues 95 to 228 is fully functional for binding to ODC, inhibiting ODC enzyme activity, and degrading ODC through AZ-mediated 26S proteasomal protein degradation [41]. Here, we report the distinctive structural elements of AZ that are separately responsible for binding and inhibition, as well as degradation, of ODC.

**Figure 7:**
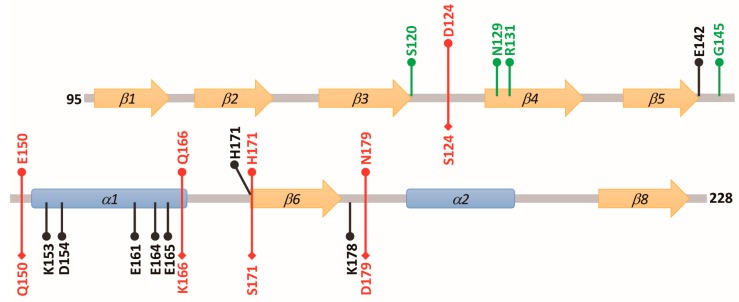
Structural elements of AZ responsible for binding, inhibition and degradation toward ODC. Black labels, amino acid residues of AZ1 critical for ODC binding and inhibition; green labels, amino acid residues of AZ1 essential for ODC degradation; red labels, amino acid residues of AZ1 and AZ3 governing the differential binding and inhibition toward ODC.

In the AZ structure, we identified at least eight amino acid residues, including Glu142, Lys153, Asp154, Glu161, Glu164, Glu165, His171, and Lys178, that are essential for binding and inhibition; these residues mainly reside the α1 helix, β5 and β6 strands and connecting loop between β6 and α2, the posterior part of AZ_95–228_ (Figure 7). In the AZ-ODC complex structure, K153, D154, E161, E164, and K178 directly interact with the respective amino acid resides of ODC; E161 and E164 are all conserved in AZ isoforms (Figure 1D), and amino acids at residues 153 and 154 demonstrate a conservative substitution (residue 153 is K in AZ1 and AZ2 but R in AZ3, and residue 154 is D in AZ1 but E in AZ2 and AZ3), ensuring the basic binding and inhibition of ODC.

We have identified the crucial factors governing the differential binding and inhibition of AZ isoforms toward ODC; these non-conservative residues (124, 150, 166, 171, and 179) determine the isoform-specific characteristics (Figure 7). Lys178 in AZ1 plays a role in ODC binding and inhibition; although this residue is not conservative (Lys in AZ1 and AZ2 but Asn in AZ3), it does not contribute to the differential binding and inhibition.

We have also identified the essential elements that determine the ODC-degradative activity of AZ. We identified at least four amino acid residues in AZ, including Ser120, Asn129, Gly137, and Gly145, which are essential for AZ-mediated ODC degradation (Figure 7). These residues mainly reside within the β3–β5 region and their connecting loops, the anterior part of AZ_95–228_ (Figure 7). In the AZ-ODC complex structure, these residues are far from the interface of AZ-ODC, presenting sites that are recognized by the proteasomal subunits. Interestingly, residues 120, 129, 137, and 145 are not conserved among the AZ isoforms, and they may present a different secondary structure preference, which may contribute to the differential ODC-degradative activity of AZ1 and AZ3. For example, Gly137, and Gly145 at AZ1 reside in the loops, which fits the secondary structure preference of glycine; the two respective amino acid residues in AZ3 are Arg, and Tyr, which may prefer to form α-helixes and β-strands. Additionally, if these sites in AZ3 are substituted with the respective residues in AZ1, the inhibitory activity of AZ3 towards ODC is reduced (Table 3). Therefore, the diverse amino acids at residues 120, 129, 137, and 145 may be one of the determinants for the differential ODC-degradative activity of AZ1 and AZ3. We tried to create a quadruple mutant of AZ3 to examine its ODC-degradative activity; unfortunately, we failed to obtain the purified quadruple mutant protein of AZ3. Therefore, the determinant for the differential ODC-degradative activity of AZ1 and AZ3 is still under investigation.

## Figures and Tables

**Table 1:** IC_50_ values for AZ_95–228_ and its mutants within the posterior part of AZ_95–228._

^1^ AZ Variants	Location	^2^ IC_50_ (μM)	^3^ Fold Change (IC_50,mutant_/IC_50,WT_)
**AZ_95–228_**	C-terminal domain	0.16 ± 0.01	1
**AZ_95–228__E142A**	β5	0.31 ± 0.02	1.93
**AZ_95–228__K153A**	α1	0.40 ± 0.10	2.5
**AZ_95–228__D154A**	α1	0.27 ± 0.06	1.69
**AZ_95–228__E161A**	α1	0.25 ± 0.06	1.56
**AZ_95–228__E164A**	α1	0.30 ± 0.06	1.88
**AZ_95–228__E165A**	α1	0.30 ± 0.07	1.88
**AZ_95–228__H171A**	β6	0.27 ± 0.09	1.69
**AZ_95–228__K178A**	Loop between β6 and α2	0.31 ± 0.05	1.94
**AZ_95–228_-3Mα1**	α1	4.13 ± 0.34	25.82
**AZ_95–228_-3Mα1_D154A**	α1	5.61 ± 0.35	35.08
**AZ_95–228_-3Mα1_E161A**	α1	6.31 ± 5.95	39.45
**AZ_95–228_-5Mα1**	α1	No Inhibition	-
**AZ_95–228_-3Mα1_E142A**	α1/β5	No Inhibition	-
**AZ_95–228_-3Mα1_H171A**	α1/β6	No Inhibition	-
**AZ_95–228_-3Mα1_K178A**	α1/loop_β6-α2	No Inhibition	-
**AZ_95–228__E142A/H171A/K178A**	β5/β6/loop_β6-α2	No Inhibition	-
**AZ_95–228_-8M**	β5/α1/β6/loop_β6-α2	No Inhibition	-

^1^ AZ_95–228_-3Mα1: AZ_95–228__K153A/E164A/E165A, AZ_95–228_-5Mα1: AZ_95–228__K153A/D154A/E161A/E164A/E165A, AZ_95–228_-8M: AZ_95–228__E142A/K153A/D154A/E161A/E164A/E165A/H171A/K178A. ^2^ All IC_50_ values were derived from fitting the inhibition curves of ODC shown in Figure 2 and Figure S2. ^3^ Fold change was the ratio of IC_50_ of the mutant versus IC_50_ of WT.

**Table 2:** Dissociation constants of human AZ_95–228_-ODC heterodimers.

AZ_95–228_-ODC dimer	^1^*K_d_*_,AZ-ODC_ (μM)	^2^ Fold Change (*K_d_*_,mutant_/*K_d_*_,WT_)
**AZ_95–228_-ODC**	1.15 ± 0.01	1
**AZ_95–228__E142A-ODC**	2.20 ± 0.02	1.9
**AZ_95–228__K153A-ODC**	1.21 ± 0.01	1.05
**AZ_95–228__D154A-ODC**	1.70 ± 0.02	1.5
**AZ_95–228__E161A-ODC**	1.11 ± 0.01	0.97
**AZ_95–228__E164A-ODC**	1.68 ± 0.01	1.46
**AZ_95–228__E165A-ODC**	1.73 ± 0.02	1.5
**AZ_95–228__H171A-ODC**	2.03 ± 0.02	1.77
**AZ_95–228__K178A-ODC**	3.03 ± 0.02	2.63
**AZ_95–228_-3Mα1-ODC**	6.58 ± 0.02	5.72
**AZ_95–228_-3Mα1_D154A**	3.39 ± 0.03	2.95
**AZ_95–228_-3Mα1_E161A-ODC**	4.97 ± 0.06	4.32
**AZ_95–228_-5Mα1-ODC**	6.81 ± 0.06	5.92
**AZ_95–228_-3Mα1_E142A-ODC**	8.86 ± 0.05	7.7
**AZ_95–228_-3Mα1_H171A-ODC**	8.55 ± 0.08	7.43
**AZ_95–228_-3Mα1_K178A-ODC**	7.67 ± 0.06	6.67
**AZ_95–228__E142A/H171A/K178A-ODC**	11.49 ± 0.6	10
**AZ_95–228_-8M-ODC**	12.44 ± 0.7	10.8

^1^ The dissociation constants (*K*_d_) of AZ_95–228_-ODC dimer were derived by globally fitting the sedimentation velocity data (Figure 3 and Figure S3) to the A + B↔AB hetero-association model in the SEDPHAT program [50]. ^2^ Fold change was the ratio of *K_d_*_,AZ-ODC_ of the mutant versus *K_d_*_,AZ-ODC_ of WT.

**Table 3:** IC_50_ values for AZ3 and its mutants.

AZ3	^1^ IC_50_ (μM)	^2^ Fold Change (IC_50,mutant_/IC_50,WT_)
**AZ3_WT**	0.61 ± 0.07	1
**AZ3_A98D**	0.67 ± 0.12	1.1
**AZ3_G99D**	0.78 ± 0.08	1.28
**AZ3_N100R**	0.66 ± 0.07	1.08
**AZ3_T106E**	1.21 ± 0.28	2
**AZ3_D112K**	0.55 ± 0.01	0.9
**AZ3_S124D**	0.50 ± 0.06	0.82
**AZ3_T126K**	0.70 ± 0.10	1.15
**AZ3_S127R**	1.02 ± 0.41	1.67
**AZ3_H129N**	0.67 ± 0.03	1.1
**AZ3_D136G**	0.72 ± 0.08	1.18
**AZ3_R137G**	1.23 ± 0.25	2.02
**AZ3_R138S**	0.81 ± 0.20	1.33
**AZ3_Y145G**	0.98 ± 0.05	1.61
**AZ3_D149P**	2.05 ± 0.43	3.36
**AZ3_Q150E**	0.48 ± 0.01	0.79
**AZ3_K166Q**	0.42 ± 0.05	0.69
**AZ3_N168R**	1.38 ± 0.57	2.26
**AZ3_S171H**	0.43 ± 0.01	0.7
**AZ3_N175C**	0.65 ± 0.04	1.07
**AZ3_Q177H**	1.34 ± 0.21	2.19
**AZ3_N178K**	0.67± 0.04	1.1
**AZ3_D179N**	0.45 ± 0.01	0.73

^1^ The IC_50_ values were derived from fitting the inhibition curves of ODC shown in Figure 5 and Figure S6. ^2^ Fold change was the ratio of the IC_50_ of the mutant versus IC_50_ of WT.

**Table 4:** IC_50_ values and dissociation constants of human AZ-ODC heterodimers for AZ1 and AZ3 variants.

AZ Protein	^1^ IC_50_ (μM)	^2^*K_d_*_,AZ-ODC_ (μM)
**AZ1_WT**	0.23 ± 0.03	0.22 ± 0.01
**AZ3_WT**	0.61 ± 0.07	1.52 ± 0.01
**AZ1_4M (D124S/E150Q/Q166K/N179D)**	0.55 ± 0.02	0.95 ± 0.03
**AZ3_4M (S124D/Q150E/K166Q/D179N)**	0.25 ± 0.01	0.50 ± 0.01
**AZ1_5M (D124S/E150Q/Q166K/H171S/N179D)**	0.49 ± 0.02	1.78 ± 0.02
**AZ3_5M (S124D/Q150E/K166Q/S171H/D179N)**	0.29 ± 0.01	0.14 ± 0.01

^1^ The IC_50_ values were derived from fitting the inhibition curves of ODC (Figure 5). ^2^ The *K_d_* values of AZ-ODC dimer were derived by globally fitting the sedimentation velocity data (Figure 6).

## Supplementary Materials

The supplementary materials are available online at https://www.mdpi.com/2218-273X/9/12/864/s1.
