# Supplementary material for: Critical Factors in Human Antizymes that Determine the Differential Binding, Inhibition, and Degradation of Human Ornithine Decarboxylase
Source: Biomolecules. 2019 Dec 12;9(12):864. doi: 10.3390/biom9120864 (PMC6995573; doi:10.3390/biom9120864)
Supplement: Supplementary file 1 [file biomolecules-09-00864-s001.pdf]

Supplementary Data

Critical factors in human antizymes that determine the differential binding, inhibition, and degradation of human ornithine decarboxylase

Ju-Yi Hsieh, Yen-Chin Liu, I-Ting Cheng, Chu-Ju Lee, Yu-Hsuan Wang, Yi-Shiuan Fang, Yi-Liang Liu, Guang-Yaw Liu, Hui-Chih Hung

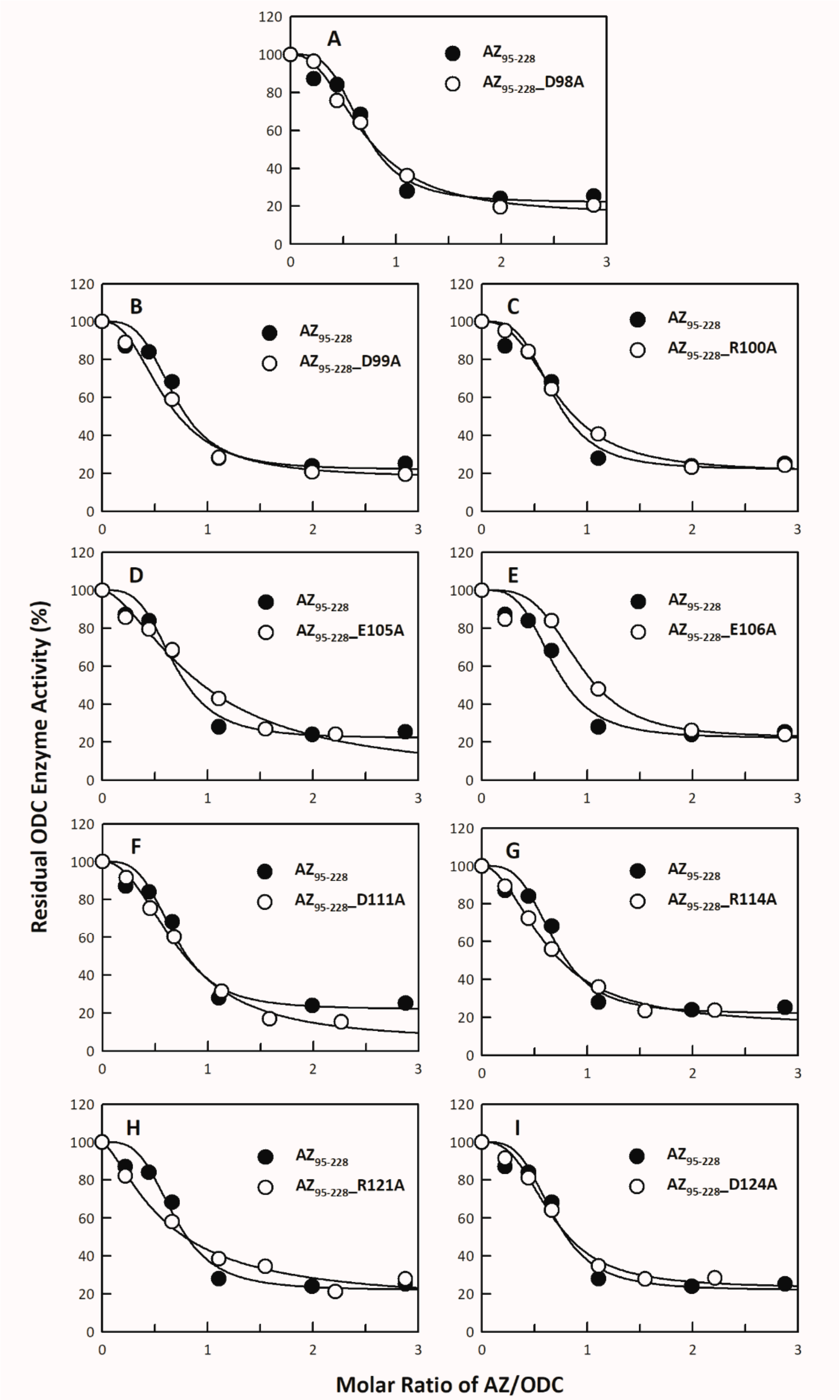

Figure S1: Inhibition plots of the ODC enzyme with single mutants of $AZ_{95\text{-}228}$ within the β1-β3 region and their connecting loops.

The enzyme activity of ODC was inhibited by various single mutants of $AZ_{95\text{-}228}$. The IC_{50} value of each single mutant of $AZ_{95\text{-}228}$ presented in Table S1 was derived by curve-fitting the inhibition plots. The molar ratio refers to $AZ_{95\text{-}228}$ versus the ODC monomer. (A) $AZ_{95\text{-}228}$_D98A, (B) $AZ_{95\text{-}228}$_D99A, (C) $AZ_{95\text{-}228}$_R100A, (D) $AZ_{95\text{-}228}$_E105A, (E) $AZ_{95\text{-}228}$_E106A, (F) $AZ_{95\text{-}228}$_D111A, (G) $AZ_{95\text{-}228}$_R114A, (H) $AZ_{95\text{-}228}$_R121A and (I) $AZ_{95\text{-}228}$_D124A.

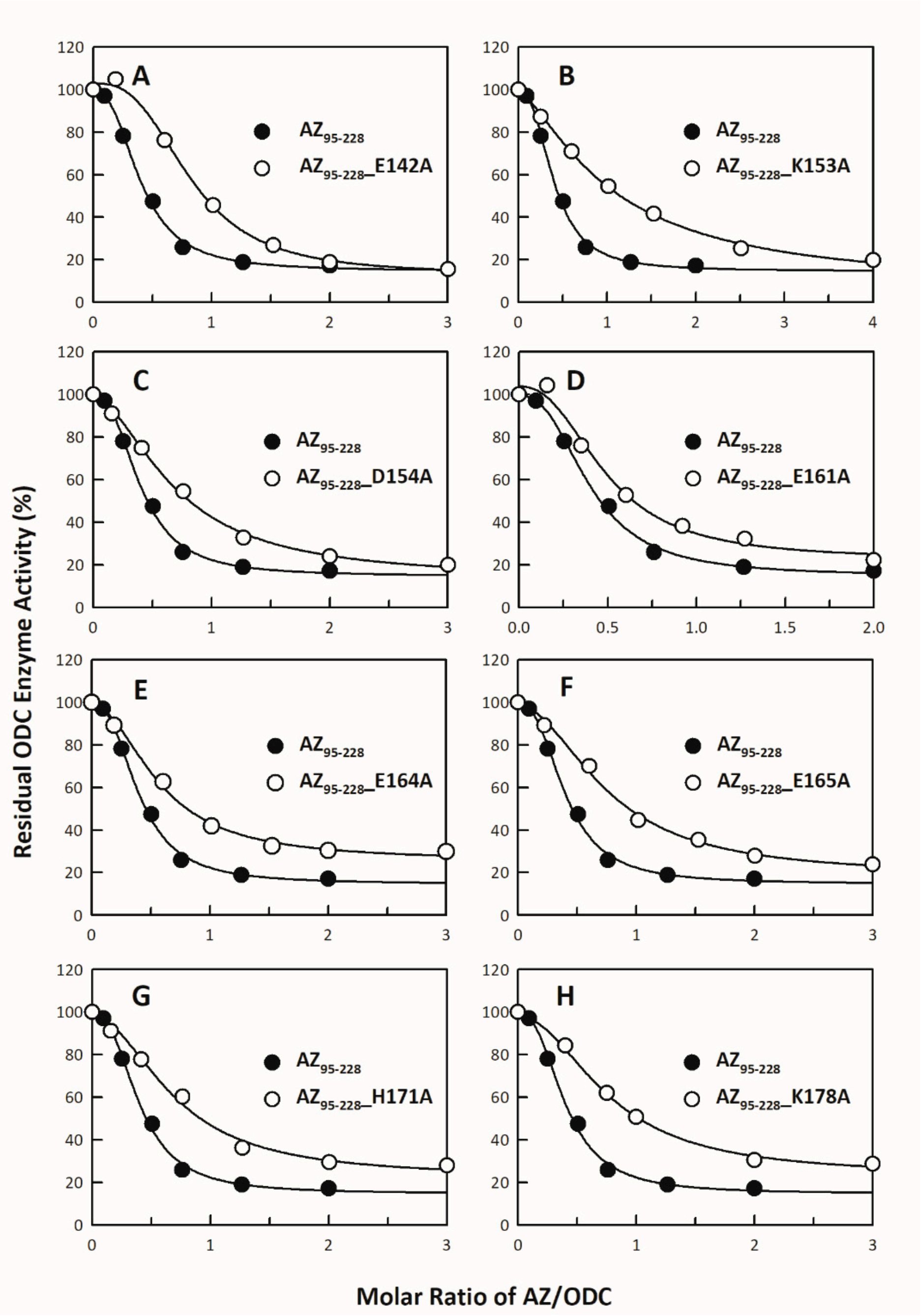

Figure S2: Inhibition plots of the ODC enzyme with single mutants within the region after β4 strand of AZ$_{95\text{-}228}$.

The enzyme activity of ODC was inhibited by various single mutants of AZ$_{95\text{-}228}$. The IC$_{50}$ values of single mutants of AZ$_{95\text{-}228}$ presented in Table 1 were derived by curve-fitting the inhibition plots. The molar ratio refers to AZ$_{95\text{-}228}$ versus the ODC monomer. (A) AZ$_{95\text{-}228}$_E142A, (B) AZ$_{95\text{-}228}$_K153A, (C) AZ$_{95\text{-}228}$_D154A, (D) AZ$_{95\text{-}228}$_E161A, (E) AZ$_{95\text{-}228}$_E164A, (F) AZ$_{95\text{-}228}$_D165A, (G) AZ$_{95\text{-}228}$_H171A, and (H) AZ$_{95\text{-}228}$_K178A.

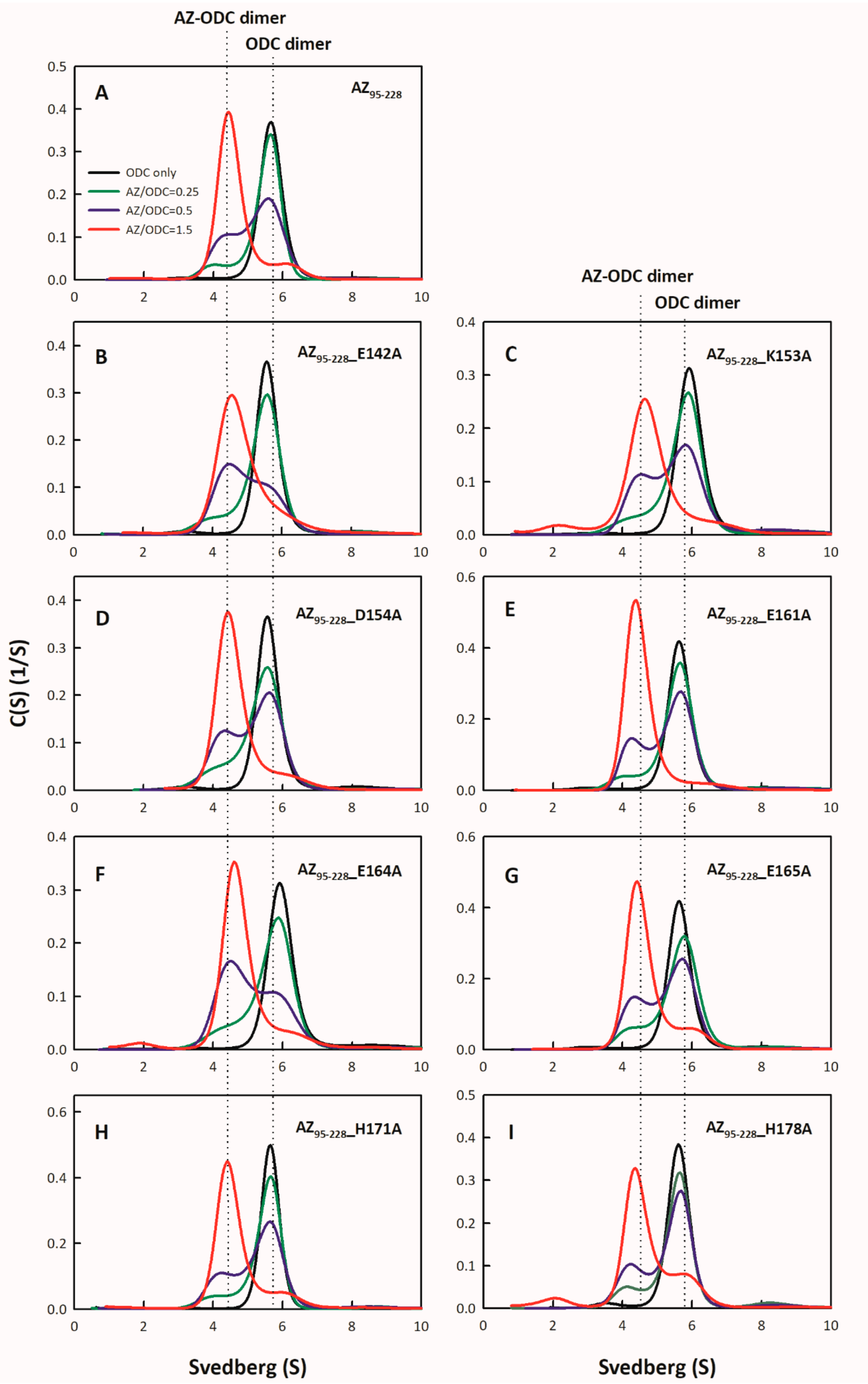

Figure S3: Plots of continuous sedimentation coefficient distributions of the single mutants of AZ$_{95\text{-}228}$-ODC.

(A) AZ$_{95\text{-}228}$-ODC, (B) AZ$_{95\text{-}228}$_E142A-ODC, (C) AZ$_{95\text{-}228}$_K153A-ODC, (D) AZ$_{95\text{-}228}$_D154A-ODC, (E) AZ$_{95\text{-}228}$_E161A-ODC, (F) AZ$_{95\text{-}228}$_E164A-ODC, (G) AZ$_{95\text{-}228}$_D165A-ODC, (H) AZ$_{95\text{-}228}$_H171A-ODC, and (I) AZ$_{95\text{-}228}$_K178A-ODC. The sedimentation velocity data for each figure were globally fitted with the SEDPHAT program to acquire K_d values for the AZ$_{95\text{-}228}$-ODC heterodimers shown in Table 2.

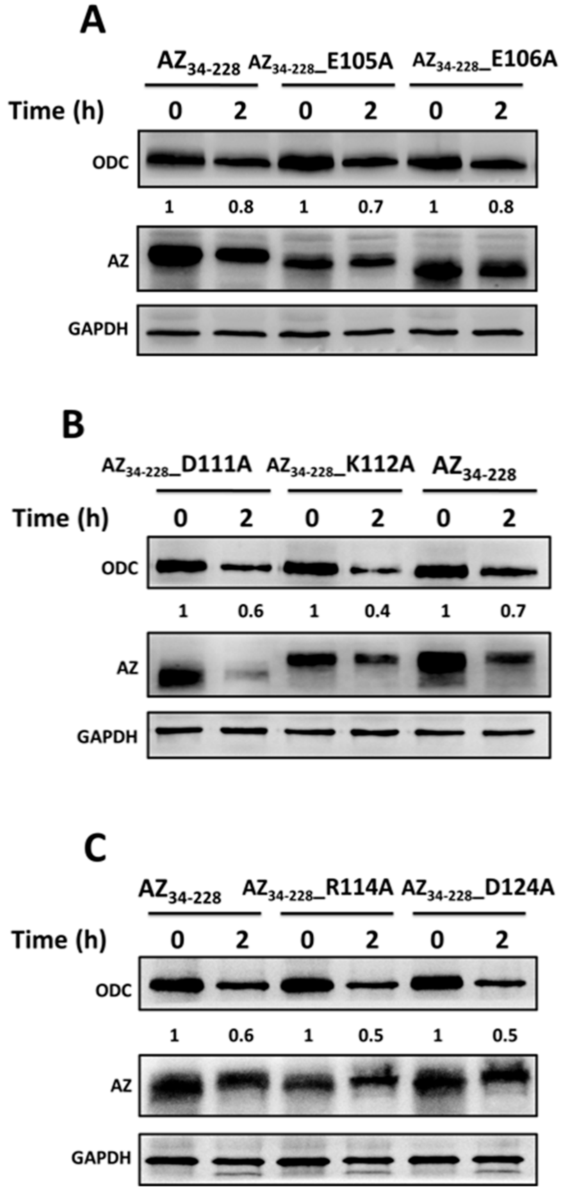

Figure S4: AZ-mediated ODC in vitro degradation with AZ$_{34-228}$ mutant peptides in the rabbit reticulocyte lysate.

ODC degradation by AZ mutants was detected by anti-ODC antibody (n=3). (A) ODC degradation with AZ$_{34-228}$, AZ$_{34-228}$_E105A and AZ$_{34-228}$_E106A, (B) ODC degradation with AZ$_{34-228}$, AZ$_{34-228}$_D111A and AZ$_{34-228}$_K112A, (C) ODC degradation with AZ$_{34-228}$, AZ$_{34-228}$_R114A and AZ$_{34-228}$_D124A. A residual amount of ODC protein at a different time was indicated under the ODC blotting gel in each figure.

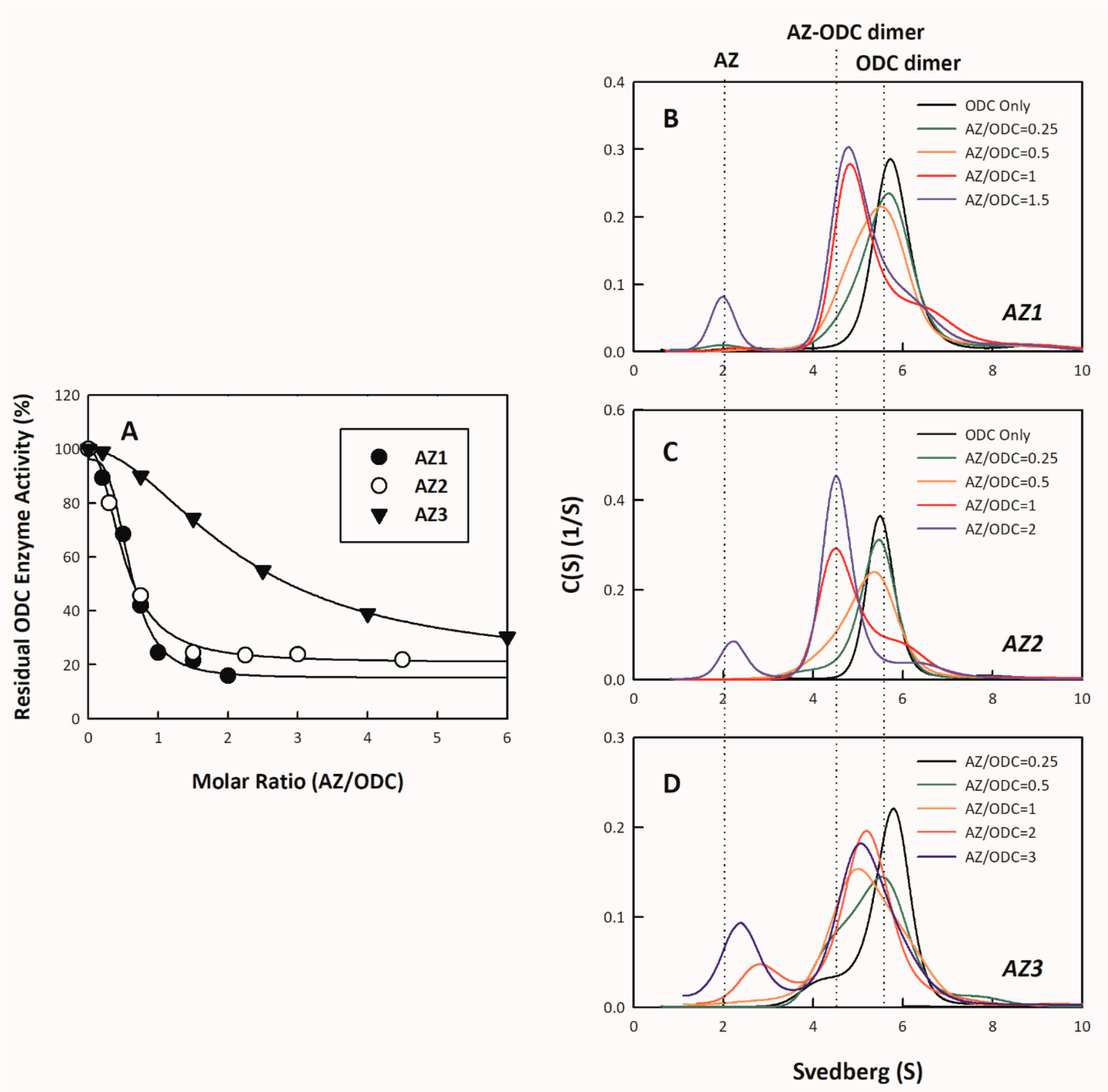

Figure S5: Binding and inhibition of AZ isoforms toward ODC.

(A) Inhibition plots of AZ1 (closed circles), AZ2 (open circles) and AZ3 (closed triangles). (B), (C) and (D) Size distribution plots of AZ1, AZ2 and AZ3, respectively. The IC_{50} values of AZ1, AZ2 and AZ3 were 0.23 μM, 0.19 μM and 0.84 μM, and the $K_{d,AZ\text{-}ODC}$ values were 0.22 μM, 0.28 μM and 0.59 μM, respectively.

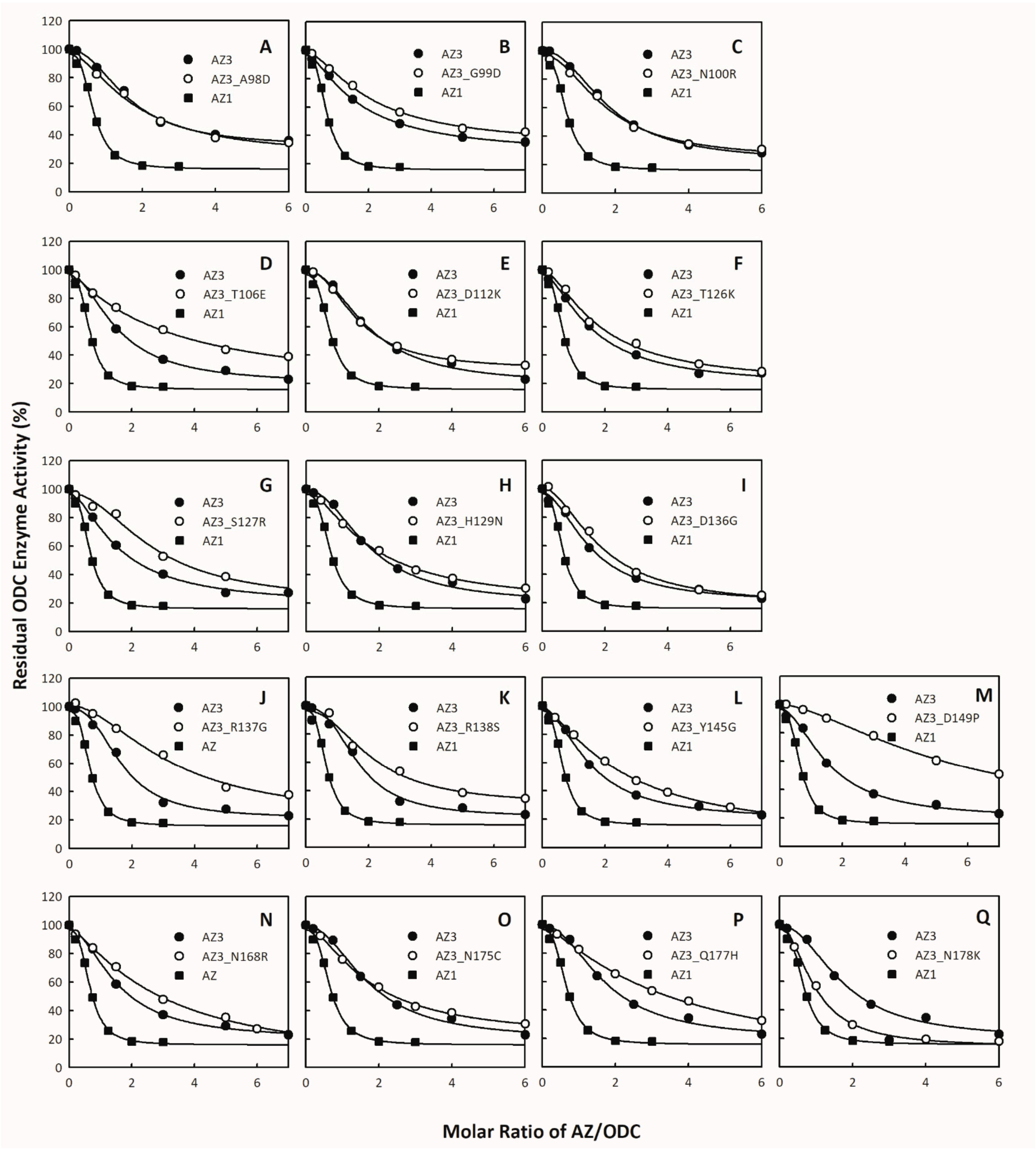

Figure S6: Inhibition plots of the ODC enzyme with single mutants of AZ3.

The enzyme activity of ODC was inhibited by various single mutants of AZ3. The IC_{50} values of single mutants of AZ3 presented in Table 3 were derived by curve-fitting the inhibition plots. The molar ratio refers to AZ3 versus the ODC monomer. (A) AZ3_A98D, (B) AZ3_G99D, (C) AZ3_N100R, (D) AZ3_T106E, (E) AZ3_D112K, (F) AZ3_T126K, (G) AZ3_S127R, (H) AZ3_H129N, (I) AZ3_D136G, (J) AZ3_R137G, (K) AZ3_R138S, (L) AZ3_Y145G, (M) AZ3_D149P, (N) AZ3_N168R, (O) AZ3_N175C, (P) AZ3_Q177H, and (Q) AZ3_N178K.

Table S1: IC_{50} values for $AZ_{95\text{-}228}$ and its mutants within the β1-β3 region and their connecting loops.

AZ Variants	Location	[1]IC_{50} (μM)	[2]Fold Change ($IC_{50,mutant}/IC_{50,WT}$)
**$AZ_{95\text{-}228}$**	C-terminal domain	0.16 ± 0.01	1
**$AZ_{95\text{-}228}$_D98A**	β1	0.18 ± 0.02	1.13
**$AZ_{95\text{-}228}$_D99A**	β1	0.17 ± 0.02	1.06
**$AZ_{95\text{-}228}$_R100A**	β1	0.19 ± 0.01	1.19
**$AZ_{95\text{-}228}$_E105A**	β2	0.19 ± 0.08	1.19
**$AZ_{95\text{-}228}$_E106A**	β2	0.20 ± 0.06	1.3
**$AZ_{95\text{-}228}$_D111A**	Loop between β2 and β3	0.19 ± 0.02	1.19
**$AZ_{95\text{-}228}$_R114A**	β3	0.16 ± 0.02	1
**$AZ_{95\text{-}228}$_R121A**	Loop between β3 and β4	0.16 ± 0.06	1
**$AZ_{95\text{-}228}$_D124A**	Loop between β3 and β4	0.17± 0.02	1.06

[1]The IC_{50} values were derived from fitting the inhibition curves of ODC shown in Figure S1.

[2]Fold change was the ratio of the IC_{50} of the mutant versus IC_{50} of WT.

Table S2: Mutagenic primers for the site-directed mutagenesis of AZ protein

AZ1 Variants	Forward Primers
**AZ1_D98A**	5'-CAGCTAACTTATTCTACTCC**GCG**GATCGGCTGAATGTAACAG-3'
**AZ1_D99A**	5'-GCTAACTTATTCTACTCCGAT**GCG**CGGCTGAATGTAACAGAGG-3'
**AZ1_R100A**	5'-CTAACTTATTCTACTCCGATGAT**GCG**CTGAATGTAACAGAGGAAC-3'
**AZ1_E105A**	5'-GATCGGCTGAATGTAACA**GCG**GAACTAACGTCCAACGAC-3'
**AZ1_E106A**	5'-GGCTGAATGTAACAGAG**GCG**CTAACGTCCAACGACAAG-3'
**AZ1_N110A**	5'-GAGGAACTAACGTCC**GCG**GACAAGACGAGGATTC-3'
**AZ1_D111A**	5'-GAACTAACGTCCAAC**GCG**AAGACGAGGATTCTC-3'
**AZ1_K112A**	5'-CTAACGTCCAACGAC**GCG**ACGAGGATTCTCAACG-3'
**AZ1_R114A**	5'-CTAACGTCCAACGACAAGACG**GCG**ATTCTCAACGTCCAGTCCAGG-3'
**AZ1_N117A**	5'-CAAGACGAGGATTCTC**GCG**GTCCAGTCCAGGCTC-3'
**AZ1_S120A**	5'-GATTCTCAACGTCCAG**GCG**AGGCTCACAGACGCC-3'
**AZ1_R121A**	5'-AGGATTCTCAACGTCCAGTCC**GCG**CTCACAGACGCCAAACGCATT-3'
**AZ1_D124A**	5'- GCCTCTACATC**GCG**ATCCCGGGCGG-3'
**AZ1_N129A**	5'-CAGACGCCAAACGCATT**GCG**TGGCGAACAGTGCTG-3'
**AZ1_R131A**	5'-CAAACGCATTAACTGG**GCG**ACAGTGCTGAGTGGC-3'
**AZ1_G136A**	5'-GCGAACAGTGCTGAGT**GCG**GGCAGCCTCTACATCG-3'
**AZ1_G137A**	5'-GAACAGTGCTGAGTGGC**GCG**AGCCTCTACATCGAGATC-3'
**AZ1_E142A**	5'-GCCTCTACATC**GCG**ATCCCGGGCGG-3'
**AZ1_G145A**	5'-CTACATCGAGATCCCG**GCG**GGCGCGCTGCCCGAG-3'
**AZ1_K153A**	5'- GCCCGAGGGGAGC**GCG**GACAGCTTTGCAG-3'
**AZ1_D154A**	5'- GAGGGGAGCAAG**GCG**AGCTTTGCAGTTC-3'
**AZ1_E161A**	5'- GCAGTTCTCCTG**GCG**TTCGCTGAGGAG-3'
**AZ1_E164A**	5'- CTGGAGTTCGCT**GCG**GAGCAGCTGCG-3'
**AZ1_E165A**	5'- GAGTTCGCTGAG**GCG**CAGCTGCGAGC-3'
**AZ1_H171A**	5'- CAGCTGCGAGCCGAC**GCG**GTCTTCATTTGCTTC-3'
**AZ1_K178A**	5'- CTTCATTTGCTTCCAC**GCG**AACCGCGAGGACA-3'
**AZ3_A98D**	5'-CTTAAAGAACTGTATTCG**GAC**GGGAACTTGACGGTG-3'
**AZ3_G99D**	5'-CTTAAAGAACTGTATTCGGCT**GAC**AACTTGACGGTGCTGGCTACT-3'
**AZ3_N100R**	5'-AAAGAACTGTATTCGGCTGGG**CGT**TTGACGGTGCTGGCTACTGAC-3'
**AZ3_T106E**	5'-GACGGTGCTGGCT**GAA**GACCCCCTGCTCCAC-3'
**AZ3_D112K**	5'-CTGACCCCCTGCTCCACCAG**AAA**CCAGTACAGTTAGACTTTCAC-3'
**AZ3_S124D**	5'-CTTTCACTTCCGCCTTACC**GAC**CAGACCTCTGCCCATTGGC-3'
**AZ3_T126K**	5'-CTTCCGCCTTACCTCCCAG**AAA**TCTGCCCATTGGCACGGCCT-3'
**AZ3_S127R**	5'-CGCCTTACCTCCCAGACC**CGT**GCCCATTGGCACGGCCTTCTC-3'
**AZ3_H129N**	5'-CTCCCAGACCTCTGCC**AAC**TGGCACGGCCTTCTC-3'
**AZ3_D136G**	5'-ATTGGCACGGCCTTCTCTGT**GGT**CGTCGACTCTTCCTGGATAT-3'
**AZ3_R137G**	5'-GCACGGCCTTCTCTGTGAC**GGT**CGACTCTTCCTGGATATCCC-3'
**AZ3_R138S**	5'-CACGGCCTTCTCTGTGACCGT**TCT**CTCTTCCTGGATATCCCATATC-3'

**AZ3_Y145G**	5'-GTTTGTGGAGATCCCG**GGT**GGTCTGCTGGCCGAT-3'
**AZ3_D149P**	5'-GATATCCCATATCAGGCCTTG**CCG**CAAGGCAACCGGGAAAGTTTG-3'
**AZ3_Q150E**	5'-CCCATATCAGGCCTTGGAT**GAA**GGCAACCGGGAAAGTTTGAC-3'
**AZ3_K166Q**	5'-CCTGGAGTACGTGGAAGAG**CAG**ACAAATGTGGACTCTGTGT-3'
**AZ3_N168R**	5'-GTACGTGGAAGAGAAGACA**CGT**GTGGACTCTGTGTTTGTGAAC-3'
**AZ3_S171H**	5'-GAGAAGACAAATGTGGAC**CAC**GTGTTTGTGAACTTCCAG-3'
**AZ3_N175C**	5'-GTGGACTCTGTGTTTGTG**TGC**TTCCAGAATGATCGG-3'
**AZ3_Q177H**	5'-GTGTTTGTGAACTTC**CAC**AATGATCGGAACGACAG-3'
**AZ3_N178K**	5'-GTTTGTGAACTTCCAG**AAA**GATCGGAACGACAGAGG-3'
**AZ3_D179N**	5'-GTTTGTGAACTTCCAGAAT**AAC**CGGAACGACAGAGGTGCCCT-3'
